# Total flavonoids of *Astragalus* Ameliorated Bile Acid Metabolism Dysfunction in Diabetes Mellitus

**DOI:** 10.1155/2021/6675567

**Published:** 2021-04-12

**Authors:** Zhe Wang, Xu-Ling Li, Kin-Fong Hong, Ting-Ting Zhao, Rui-Xue Dong, Wei-Ming Wang, Yun-Tong Li, Gui-Lin Zhang, Jing Lin, Ding-Kun Gui, You-Hua Xu

**Affiliations:** ^1^Faculty of Chinese Medicine, State Key Laboratory of Quality Research in Chinese Medicine, Macau University of Science and Technology, Taipa, Macao, China; ^2^School of Pharmacy, State Key Laboratory of Quality Research in Chinese Medicine, Macau University of Science and Technology, Taipa, Macao, China; ^3^Guangdong Provincial Hospital of Chinese Medicine-Zhuhai Hospital, Zhuhai, Guangdong, China; ^4^Department of Nephrology, Shanghai Jiao Tong University Affiliated to Sixth People's Hospital, Shanghai 200233, China; ^5^Department of Endocrinology, Zhuhai Hospital of Integrated Traditional Chinese and Western Medicine, Zhuhai, China

## Abstract

*Astragalus Radix* is one of the common traditional Chinese medicines used to treat diabetes. However, the underlying mechanism is not fully understood. Flavones are a class of active components that have been reported to exert various activities. Existing evidence suggests that flavones from *Astragalus Radix* may be pivotal in modulating progression of diabetes. In this study, total flavones from *Astragalus Radix* (TFA) were studied to observe its effects on metabolism of bile acids both *in vivo* and *in vitro*. C57BL/6J mice were treated with STZ and high-fat feeding to construct diabetic model, and HepG2 cell line was applied to investigate the influence of TFA on liver cells. We found a serious disturbance of bile acids and lipid metabolism in diabetic mice, and oral administration or cell incubation with TFA significantly reduced the production of total cholesterol (TCHO), total triglyceride, glutamic oxalacetic transaminase (AST), glutamic-pyruvic transaminase (ALT), and low-density lipoprotein (LDL-C), while it increased the level of high-density lipoprotein (HDL-C). The expression of glucose transporter 2 (GLUT2) and cholesterol 7*α*-hydroxylase (CYP7A1) was significantly upregulated on TFA treatment, and FXR and TGR5 play pivotal role in modulating bile acid and lipid metabolism. This study supplied a novel understanding towards the mechanism of *Astragalus Radix* on controlling diabetes.

## 1. Introduction

Although a giant leap has been made in the prevention and treatment of diabetes mellitus (DM), the number of patients continues to rise. The International Diabetes Federation estimates that the DM patients will increase to 700 million by 2045 [1].

Studies have proved that bile acids are closely related to DM and glycolipid metabolism [2]. Liver is the main organ that secretes bile acids, and hepatoenteral circulation of bile acids plays an important role in maintaining the homeostasis of glycolipids in the body [3, 4]. It has been well demonstrated that metabolic changes of bile acid may induce glycolipidemia via FXR and TGR5 pathways [5, 6]. Alternatively, long-lasting hyperglycemia will influence liver function and secretion of bile acids [7]. Therefore, modulating metabolism of bile acids is believed to be a potential target on preventing DM.

Studies concerning effects of *Astragalus Radix* (*Huang Qi* in Chinese) on DM are abundant. The active components of *Astragalus Radix* mainly include polysaccharide, astragalosides, and flavones [8, 9]. Previously, we have reported that flavones calycosin and calycosin-7-O-*β*-D-glucopyranoside could ameliorate vascular endothelial cell dysfunction under diabetic settings [10–12]; moreover, calycosin also possessed protective effects on hepatocyte function [13]. Besides that, other research groups have also reported that total flavonoids of *Astragalus Radix* (TFA) has anti-inflammatory effects [14, 15]. Recently, it was reported that *Astragalus Radix* decoction has a regulatory effect on bile acid [16]. However, the role and mechanism of TFA in the metabolism of bile acids still need to be elucidated. For this aim, we designed this study.

## 2. Materials and Methods

### 2.1. Materials

The TFA was provided by Chengdu Pusi Biotechnology, Co., Ltd. (Chengdu, Sichuan, China). Metformin was purchased from GBCBIO technology (Guangzhou, Guangdong, China). Insulin was provided by Yuanye Bio-Technology Co., Ltd. (Shanghai, China). Lipopolysaccharide (LPS) was purchased from Sigma (St. Louis, MO, USA). ELISA kits for measuring total bile acid (TBA), total cholesterol (TCHO), total triglyceride (TG), glutamic oxalacetic transaminase (AST), glutamic-pyruvic transaminase (ALT), high-density lipoprotein (HDL-C), and low-density lipoprotein (HDL-C) were purchased from Jiancheng (Nanjing, Jiangsu, China). Primary antibodies for apical sodium-dependent bile acid transporter (ASBT) were purchased from PL Laboratories (Richmond, Canada). CYP7A1 and FXR were purchased from Santa Cruz (Dallas, TX, USA). TGR5 was provided by Abcam (Cambridge, MA, USA). GLUT2 was derived from Bioss (Beijing, China). Other reagents are from commercial sources.

### 2.2. Animals

The NIH (U.S. National Institutes of Health publication no. 85–23, revised 1996) Guide for the Care and Use of Laboratory Animals was followed, and the study was approved by Macau University of Science and Technology. C57BL/6J mice were provided from Guangdong Medical Laboratory Animal Center. All mice were housed in an animal house with a 12 h daylight cycle at 25°C. Before drug intervention, all mice were fed adaptively for 7 days. The animals were fed a high-fat diet (15% protein, 43% carbohydrate, 42% fat; obtained from Guangdong Medical Experimental Animal Center) for 8 weeks and then treated with streptozotocin (50 mg/kg per day) for 4 days to make the model. The animals were randomly divided into groups as follows: (1) normal control group (*n* = 6, animals were fed normal chow); (2) DM model group (*n* = 6), where animals were given streptozotocin; (3) intervention group where DM animal models were orally treated with low TFA (L-TFA, 5 mg/kg per day, *n* = 6), medium TFA (M-TFA, 25 mg/kg per day, *n* = 6), and high TFA (H-TFA, 50 mg/kg per day, *n* = 6); (4) positive control group where animals were orally administrated metformin (Met, 0.15 g/kg per day, *n* = 6). At the end of the experiment, liver was collected for histological or biochemical study.

### 2.3. Cell Lines and Cell Culture

HepG2 cells were derived from American Type Culture Collection (Manassas, VA, USA). All cells were cultured using MEM medium (Gibco) supplemented with 10% fetal bovine serum, 100 U/mL penicillin, and 100 mg/mL streptomycin at 37°C in 5% CO_2_ incubator.

### 2.4. MTT Assay for Cell Viability

The HepG2 cells were inoculated into a 96-well cell culture plate with 1 × 10^5^ cells per well. Different concentrations of TFA (0–40 *μ*g/mL) were added to the cells. After 24 h, MTT (3-(4,5-dimethylth-iazol-2-yl)-2,5-diphenyltetrazolium bromide)) (0.5 mg/ml) was added to the culture system and cultured in dark for 4 h. Thyroid crystal was dissolved with 10% SDS (sodium dodecyl sulfate). Cell activity was analyzed at 595 nm using a plate reader (Molecular Devices, USA).

### 2.5. Histological Analysis

Liver slices were observed for hematoxylin-eosin (H&E) and periodic acid-schiff (PAS) staining. The liver slices were fixed within 4% paraformaldehyde over 24 h; after being paraffinized, sections (0.4 *μ*m) were stained with H&E or PAS staining solution according to the standard procedure. Finally, the slices were covered with coverslip and imaged using the Leica DM2500 Fluorescence Microscope Imaging System (Leica, Germany).

### 2.6. Western Blot Analysis

Liver tissue was lysed in 1 × RIPA buffer with a protease inhibitor cocktail and phosphatase inhibitor. Total protein was quantified by using the Bio-Rad Protein Assay (USA). Samples were separated by 10% SDS-polyacrylamide gels and then transferred to polyvinylidene difluoride (PVDF) membranes (EMD Millipore, MA, USA). After blocking with 5% BSA in TBST buffer, the PVDF membrane was incubated with primary antibodies including anti-GAPDH antibody (1 : 1000), anti-CYP7A1 antibody (1 : 800), anti-FXR antibody (1 : 800), anti-TGR5 antibody (1 : 1000), or anti-GLUT2 antibody (1 : 1000) overnight at 4°C. After that, the membranes were washed with TBST buffer for three times and then further incubated with secondary antibody (Invitrogen, USA) for 1 h at room temperature. Protein detection was carried out by the Odyssey CLx Imaging System (Li-COR Biosciences, Belfast, ME, USA).

### 2.7. Immunofluorescence Assay

Immunofluorescence assay was performed as previously described [17]. HepG2 cells in the exponential phase were seeded on glass slides, normal MEM was used in normal group, Ins + LPS (insulin, 10^−7^ mol/L; LPS: 1 *μ*g/ml) was added in DM group, Ins + LPS + TFA was added in TFA group, and metformin (Met, 0.5 mM) was added in positive control group [18, 19]. Twenty-four hours later, cells were treated with 4% paraformaldehyde for 30 min. After being blocked with 5% BSA, cells were incubated with antibodies including anti-GLUT2 (1 : 200), anti-FXR (1 : 200), anti-CYP7A1 (1 : 200), anti-TGR5 (1 : 200), or anti-ASBT (1 : 200) at 4°C overnight and then were further incubated with FITC- or CY3 -conjugated secondary antibody. The nucleus was stained with DAPI. Finally, the cells were observed under a confocal laser scanning microscopy (Leica TCS SP8, Germany), and the fluorescent density was determined by Image-J software.

### 2.8. Statistical Analysis

Figure preparation and statistical analysis were carried out using GraphPad Prism 7.00 software (GraphPad Software Inc., CA, USA). Fluorescent images were processed with open-source software Image-J. All data were obtained from more than three independent repeated experiments. All data that fit into the normal distribution were expressed as mean ± standard deviation (SD), and the difference among groups was analyzed by one-way ANOVA method. Significance was accepted at *p* < 0.05 or less.

## 3. Results

### 3.1. TFA Ameliorated Liver Injury and Regulated Blood Glucose in Diabetic Mice

**Figure 1:**
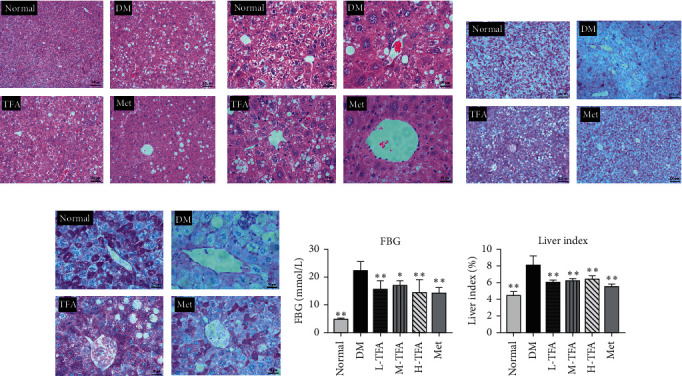
Effects of total *Astragalus* flavones (TFA) on blood glucose and liver histology in diabetic mice. H&E staining of liver (magnification: (a) 100x and (b) 400x). PAS staining of liver (magnification: (c) 100x and (d) 400x). (e) Fasting blood glucose (FBG) test. (f) Liver index; liver index = (liver weight/body weight) %. (*n* = 6). Met: metformin. ^*∗*^*p* < 0.05; ^*∗∗*^*p* < 0.01 vs. DM group.

The level of fasting blood glucose (FBG) is a sensitive parameter that reflects control of diabetes. As shown in Figure 1(e), TFA administration significantly decreased FBG in diabetic mice, suggesting definite effect of TFA on ameliorating progression of diabetes.

The liver plays a pivotal role in modulating the level of blood glucose. The H&E staining and PAS staining images (Figures 1(a)–1(d)) show that, compared with the normal group, steatosis was obviously observed in T2DM mice; moreover, the content of glycogen in hepatocytes was significantly decreased as observed by PAS staining (Figures 1(c) and 1(d)). Alternatively, TFA preserves lobular structure, maintains cellular morphology, and improves intracellular glycogen levels. The liver index was also reduced in mice treated with TFA (Figure 1(f)). These results suggest that TFA administration in T2DM mice preserved the liver function.

### 3.2. Effect of TFA on Serum Lipid Profile

**Figure 2:**
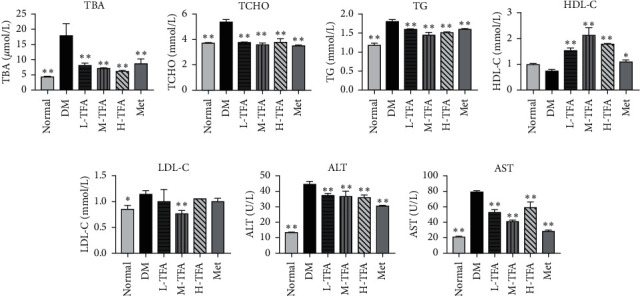
Total *Astragalus* flavones (TFA) improved lipid metabolism in mice. Serum levels of (a) total bile acid (TBA), (b) total cholesterol (TCHO), (c) total triglyceride (TG), (d) high-density lipoprotein (HDL-C), (e) low-density lipoprotein (LDL-C), (f) glutamic-pyruvic transaminase (ALT), and (g) glutamic oxalacetic transaminase (AST) were determined by kits (*n* = 6). Met: metformin. ^*∗*^*p* < 0.05; ^*∗∗*^*p* < 0.01 vs. DM group.

To investigate the influence of TFA on liver function, serum lipid profile is measured. As shown in Figures 2(a)–2(c) and Figures 2(e)–2(g), serum levels of TBA, TG, TC, LDL-C, ALT, and AST in diabetic mice were significantly increased compared with normal control, while level of HDL-C was decreased in diabetic mice, but no statistical significance was found compared with normal control (Figure 2(d)). Expectedly, administration with TFA significantly ameliorated lipid metabolism damage and restored liver function compared with diabetic mice. The most significant effect was observed in medium TFA (M-TFA) group. These results indicated that TFA can not only reduce the cholesterol level in diabetic mice but also reverse the elevated serum bile acid level in diabetic mice.

### 3.3. TFA Improved Glucose and Lipid Metabolism in the Liver of Diabetic Mice

**Figure 3:**
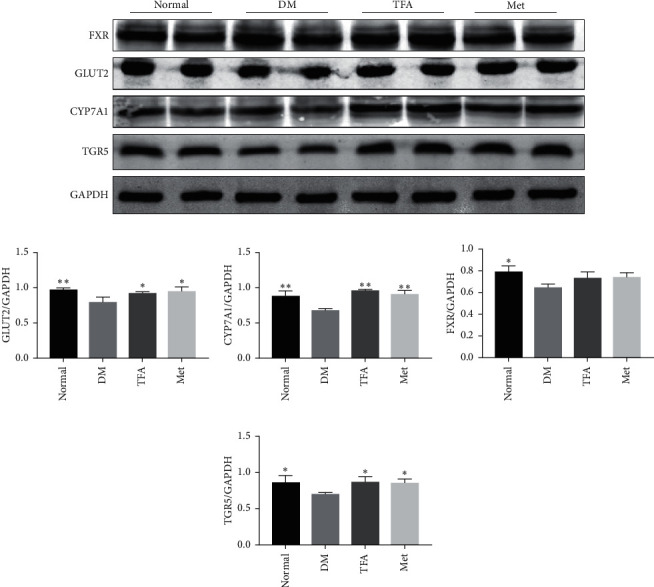
TFA increased the expression of proteins related to glucose and lipid metabolism under diabetic settings. (a) WB detection for proteins expression. Quantitative analysis of (b) GLUT2, (c) CYP7A1, (d) FXR, and (e) TGR5. Glyceraldehyde-3-phosphate dehydrogenase (GAPDH) was used as an internal control (*n* = 3). ^*∗*^*p* < 0.05; ^*∗∗*^*p* < 0.01 vs. DM group.

Existing studies have found that proteins that regulate lipid and glucose metabolism are significantly inhibited under diabetic settings, such as glucose transporter 2 (GLUT2), cholesterol 7 alpha-hydroxylase (CYP7A1), G-protein-coupled bile receptor (TGR5), and Farnesyl X receptor (FXR) [20, 21]. To study the mechanism of TFA on bile acid metabolism, their protein expression was determined by western blot. As shown in Figure 3, TFA significantly increased the expressions of GLUT2 (Figure 3(b)), CYP7A1 (Figure 3(c)), and TGR5 (Figure 3(e)) compared with those in the diabetic model group; FXR was also increased by administration of TFA, but no statistical significance was observed (Figure 3(d)).

### 3.4. TFA Improves Lipid Metabolism in HepG2 Cells

**Figure 4:**
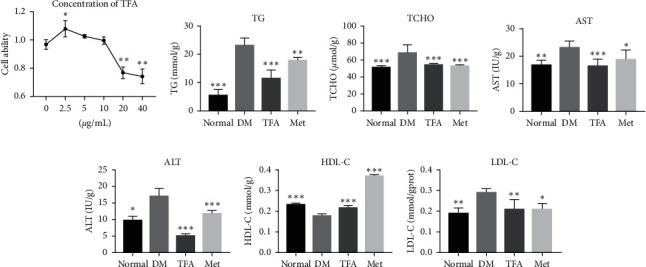
Total *Astragalus* flavones (TFA) regulated lipid metabolism in HepG2 cells. (a) MTT assay was used to evaluate the influence of TFA on cell viability. The effects of TFA on the biochemical indexes including (b) total triglyceride (TG), (c) total cholesterol (TCHO), (d) glutamic oxalacetic transaminase (AST), (e) glutamic-pyruvic transaminase (ALT), (f) high-density lipoprotein (HDL-C), and (g) low-density lipoprotein (LDL-C) were detected by the kits according to the manufacturers' protocol (*n* = 6). Met: metformin. *∗p* < 0.05; *∗∗p* < 0.01; *∗∗∗p* < 0.001 vs. DM group.

To verify the aforementioned findings, effects of TFA on HepG2 cells were further studied. MTT method was used to preliminarily detect the influence of TFA on cell viability. As shown in Figure 4(a), low concentration of TFA slightly increased the viability of HepG2 cells, but cell viability toxicity was observed once the concentration exceeds 10 *μ*g/ml; therefore, we applied “10 *μ*g/ml” in the following study.

Lipid metabolism of HepG2 cell was determined by kits. In line with findings in animals, TG, TCHO, AST, ALT, and LDL-C were significantly increased and HDL-C was decreased in diabetic mice, while TFA incubation significantly increased HDL-C and decreased other parameters (Figures 4(b)–4(g)).

### 3.5. TFA Modulated Expression of GLUT2 and Proteins Regulating Cholic Acid Metabolism in HepG2 Cells

**Figure 5:**
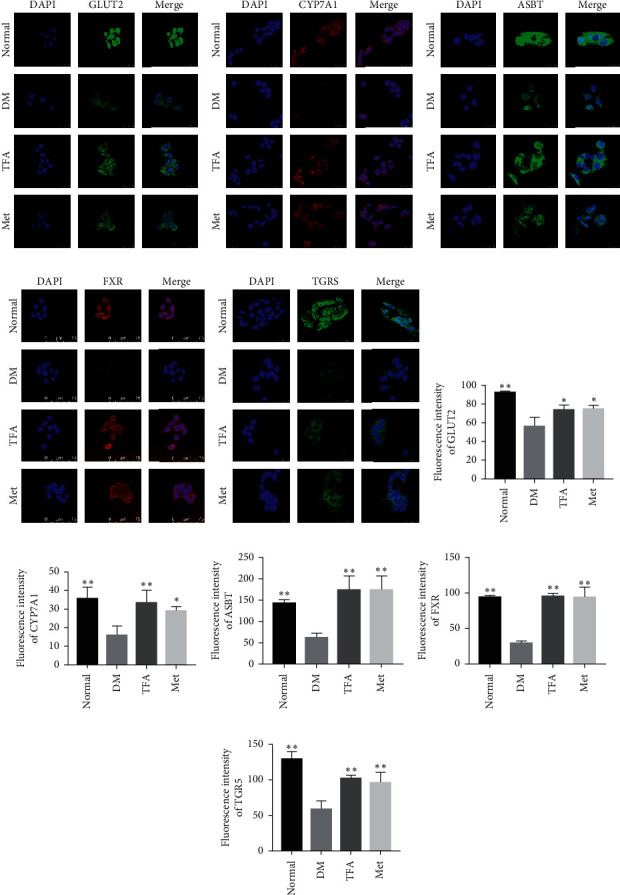
Effects of total *Astragalus* flavones (TFA) on lipid metabolism related proteins in HepG2 cells. Expressions of (a) GLUT2, (b) CYP7A1, (c) ASBT, (d) FXR, and (e) TGR5 were determined by immunofluorescence assay under laser scanning confocal microscope (magnification: 800x), and relative fluorescence intensities of (f) GLUT2, (g) CYP7A1, (h) ASBT, (i) FXR, and (j) TGR5 were determined by Image-J software. DAPI (4′,6-diamidino-2-phenylindole) was used to stain the nucleus (*n* = 3). Met: metformin. *∗p* < 0.05; *∗∗p* < 0.01 vs. DM group.

Glucose transporters play pivotal roles in mediating hepatocyte glucose uptake. By immunofluorescence assay, we found the expression of GLUT2 was significantly decreased in DM model while TFA inhibited this decrement (Figures 5(a) and 5(g)).

CYP7A1 is an enzyme that plays an important role in mediating cholesterol metabolism. In the present study, we observed that CYP7A1 was also located on HepG2 cell membranes (Figure 5(b)); moreover, DM significantly inhibited its expression (*p* < 0.01 vs. normal), and this reduction was reversed by TFA administration (*p* < 0.01 vs. DM) (Figure 5(f)).

The apical sodium-dependent bile acid transporter (ASBT) in small intestinal epithelial cells is reabsorbed into ileum cells [22], and FXR and TGR5 play key roles in cholesterol balance, fat absorption, and bile acid synthesis. In this study, we found that the reduced expression of ASBT, FXR, and TGR5 in diabetic model was significantly reversed by addition of TFA (Figures 5(c)–5(e) and 5(h)–5(j)).

## 4. Discussion

Bile acids are synthesized by hepatocytes and play a pivotal role in the digestion and absorption of lipids [23]. Recent findings have indicated its role in modulating glucose metabolism and controlling progression of diabetes. *Astragalus Radix* is a common Chinese medicine used to treat diabetes [26], and a report has demonstrated that *Astragalus Radix* can regulate blood lipid and blood glucose levels in HFD-fed mice [27]. Previously, we found active flavonoid components of *Astragalus Radix* have protective effects against diabetic damage *in vitro* [10–13], but its influence on bile acid metabolism was not studied. In the present study, we found that TFA could modulate metabolism of bile acid both *in vivo* and *in vitro*, and the potential mechanism was also studied.

The liver plays a pivotal role in modulating metabolism. With the increasing understanding of the mechanism of T2DM, the role of bile acid in disease progression has attracted scholars' interest. Within the organism, bile acids are recollected in the intestine by ASBT and transported back to the liver in a process known as the hepatointestinal circulation of bile acids. Several types of bile acid receptors have been found, one of which is the nuclear receptor, the representative of which is FXR [24]; the other group is g-protein-coupled membrane receptors, of which the representative is TGR5 [25]. Therefore, expression and activation of FXR and TGR5 will significantly influence metabolism of bile acids.

The role of bile acids in progression of diabetes has been increasingly recognized. It is found that activation of TGR5 can promote the secretion of insulin GLP-1, improve the function of pancreatic tissues, promote insulin sensitivity [28], and inhibit inflammation [29]. A rise in cholic acid within the body can activate TGR5, and binding between bile acid and TGR5 will further activate adenylate cyclase pathway and increase AMP content [30]. FXR receptor was the first bile acid receptor discovered. It was reported that cholesterol within the liver is digested by CYP7A1 to become bile acid, and the latter will be reabsorbed by ASBT in the ileum. Binding and activation of FXR-FGF19 will further inhibit the activity of CYP7A1 by activating the MAP kinase pathway, thereby inhibiting bile acid synthesis [31, 32]. Therefore, TGR5 and FXR are pivotal in modulating both genesis and metabolism of bile acid. In the present study, we found that the expressions of CYP7A1, ASBT, TGR5, and FXR were significantly reduced under diabetic settings, suggesting bile acid was inhibited in the whole process of its metabolism, and administration with TFA significantly improves its generation and metabolism.

The negative feedback of bile acid hepatointestinal circulation is mediated by small molecule heterodimer partner (SHP), which is the downstream target of FXR [34]. It was found that the activation of FXR will induce SHP-inhibited expression of CYP7A1 and ASBT in liver cells [35, 36]. Binding between SHP and transcription factor LRH-1 will regulate the expression of CYP7A1 in that LRH-1 inhibition will result in the decreased expression of CYP7A1 [37]. Studies have demonstrated that activation of FXR can promote lipoprotein metabolism [38] and reduce plasma triglyceride and cholesterol levels [39]. Moreover, the agonist of FXR was also shown to inhibit hepatic gluconeogenic genes such as glucose-6-phosphatase and increase liver glycogen synthesis [40]. Our present findings are in line with previous reports that state that a serious disturbance of lipid and bile acid metabolism was observed in diabetic mice, and administration with TFA significantly increased FXR expression and restored lipid metabolism. It has been well recognized that elevated TCHO, TG, AST, ALT, and LDL-C levels and decreased HDL-C levels are manifestations of hepatocyte injury [41, 42]. Our present findings obviously suggest that TFA have protective effects against progression of diabetes.

In conclusion, we found in the present study that TFA ameliorated lipid and bile acid metabolism under diabetic settings, and regulation of FXR and TGR5 should contribute to its effects. Our present findings provide a new understanding on mechanism of *Astragalus Radix* against progression of diabetes.

## Data Availability

The data used to support the results of this study are included within the manuscript and may be obtained from the corresponding author upon reasonable request.
